# Potential biomarkers of spinal dural arteriovenous fistula: C4BPA and C1QA

**DOI:** 10.1186/s12974-022-02522-x

**Published:** 2022-06-22

**Authors:** Yinqing Wang, Yongjie Ma, Chengbin Yang, Xiahe Huang, Kun Yang, Fei Lan, Jingxuan Fu, Zihao Song, An Tian, Yueshan Feng, Tianqi Tu, Haifeng Li, Tao Hong, Yingchun Wang, Hongqi Zhang

**Affiliations:** 1 Department of Neurosurgery, Xuanwu Hospital, Capital Medical University, No 45 Changchun Street, Xicheng District, Beijing, 100053 China; 2 China International Neuroscience Institute (China-INI), Xuanwu Hospital, Capital Medical University, Beijing, China; 3 State Key Laboratory of Molecular Developmental Biology, Institute of Genetics and Developmental Biology, Chinese Academy of Sciences, No.1 West Beichen Rd., Beijing, 100101 China; 4 Department of Evidence-Based Medicine, Xuanwu Hospital, Capital Medical University, No 45 Changchun Street, Xicheng District, Beijing, China; 5 Department of Anesthesiology, Xuanwu Hospital, Capital Medical University, No 45 Changchun Street, Xicheng District, Beijing, China; 6 Department of Clinical Laboratory, Xuanwu Hospital, National Clinical Research Center for Geriatric Diseases, Capital Medical University, Beijing, China; 7 Department of Neurology, Xuanwu Hospital, Capital Medical University, No 45 Changchun Street, Xicheng District, Beijing, China

**Keywords:** Spinal dural arteriovenous fistula, Venous hypertension myelopathy, C4BPA, C1QA, Biomarkers

## Abstract

**Background and purpose:**

A major challenge in spinal dural arteriovenous fistula (SDAVF) is timely diagnosis, but no specific predictive biomarkers are known.

**Methods:**

In the discovery cohort (case, *n* = 8 vs. control, *n* = 8), we used cerebrospinal fluid (CSF) and paired plasma samples to identify differentially expressed proteins by label-free quantitative proteomics. Further bioinformatics enrichment analyses were performed to screen target proteins. Finally, it was validated by ELISA in two of the new cohorts (case, *n* = 17 vs. control, *n* = 9), and univariate analysis, simple linear regression, and receiver operator characteristic (ROC) curve analysis were performed to evaluate the diagnostic potential.

**Results:**

In the discovery cohort, the most overexpressed proteins were APOB and C4BPA in CSF samples of patients. The GO/KEGG enrichment analysis indicated that the upregulated proteins were mainly involved in the acute inflammatory response and complement activation. Hub-gene analysis revealed that APP might be the key protein in the molecular interaction network. In the validation cohort, C4BPA and C1QA were significantly overexpressed in the CSF of patients, averaging 3046.9 ng/ml and 2167.2 ng/ml, respectively. Simple linear regression demonstrated that levels of C1QA and C4 were positively correlated with total protein in CSF (*R*^2^ = 0.8021, *p* = 0.0005; *R*^2^ = 0.7447, *p* = 0.0013). The areas under the ROC curves of C4BPA and C1QA were 0.86 and 1.00, respectively.

**Conclusions:**

This study was the first to identify C4BPA and C1QA as potential biomarkers for the diagnosis of SDAVF and revealed that complement pathway activation might be one of the molecular mechanisms for venous hypertension myelopathy.

**Supplementary Information:**

The online version contains supplementary material available at 10.1186/s12974-022-02522-x.

## Introduction

Spinal dural arteriovenous fistula (SDAVF) is a rare myelopathy with a 5–10/million annual incidence rate [1, 2]. As the most common spinal arteriovenous malformation, it accounts for approximately 70% of all spinal vascular diseases [3]. Considering the ageing population and deeper recognition of this disease, the incidence of SDAVF is increasing annually [1]. It occurs more commonly in males over 30 years old, and the thoracolumbar junction is the most common site of the fistula. The main pathological mechanism of SDAVF is related to chronic and progressive spinal injury due to venous hypertension myelopathy (VHM) [4, 5]. Patients usually had progressive flaccid paraplegia and sensory dysfunction with or without disturbance of urination and defecation.

Most patients are preliminarily diagnosed by magnetic resonance imaging (MRI), which typically shows abnormal flow void vessels over the surface of the spinal cord and intramedullary T2 hyperintense with spinal swelling. Unfortunately, MRI is occasionally atypical and neglected for patients in the early stage [6]. Some patients have neither abnormal flow voids nor intramedullary T2 hyperintensity. In a large cohort study involving 153 cases, approximately 20% of patients had no obvious abnormal vessels on MRI scans [7]. Moreover, some patients underwent lumbar puncture more than once and laboratory examinations of cerebrospinal fluid (CSF) merely showed slightly elevated protein levels. Taken together, these nonspecific observations resulted in misdiagnosis as acute transverse myelitis or demyelination diseases. Patients who present with acute or subacute onset might be treated with steroids which further exacerbates symptoms [8]. Digital subtraction angiography (DSA) is currently the gold standard for diagnosis of SDAVF, but cost, invasive risk, and the requirement for specialized training limits its practical application.

In summary, it is imperative to develop a new screening strategy for SDAVF, but there is no reliable diagnostic biomarker to date. In this study, we sought to explore potential biomarkers of SDAVF by combining the proteomics approach with antibody validation, which might provide a new perspective towards diagnosis and pathophysiology of the disease.

## Methods

### Patient recruitment and follow-up

**Fig. 1:**
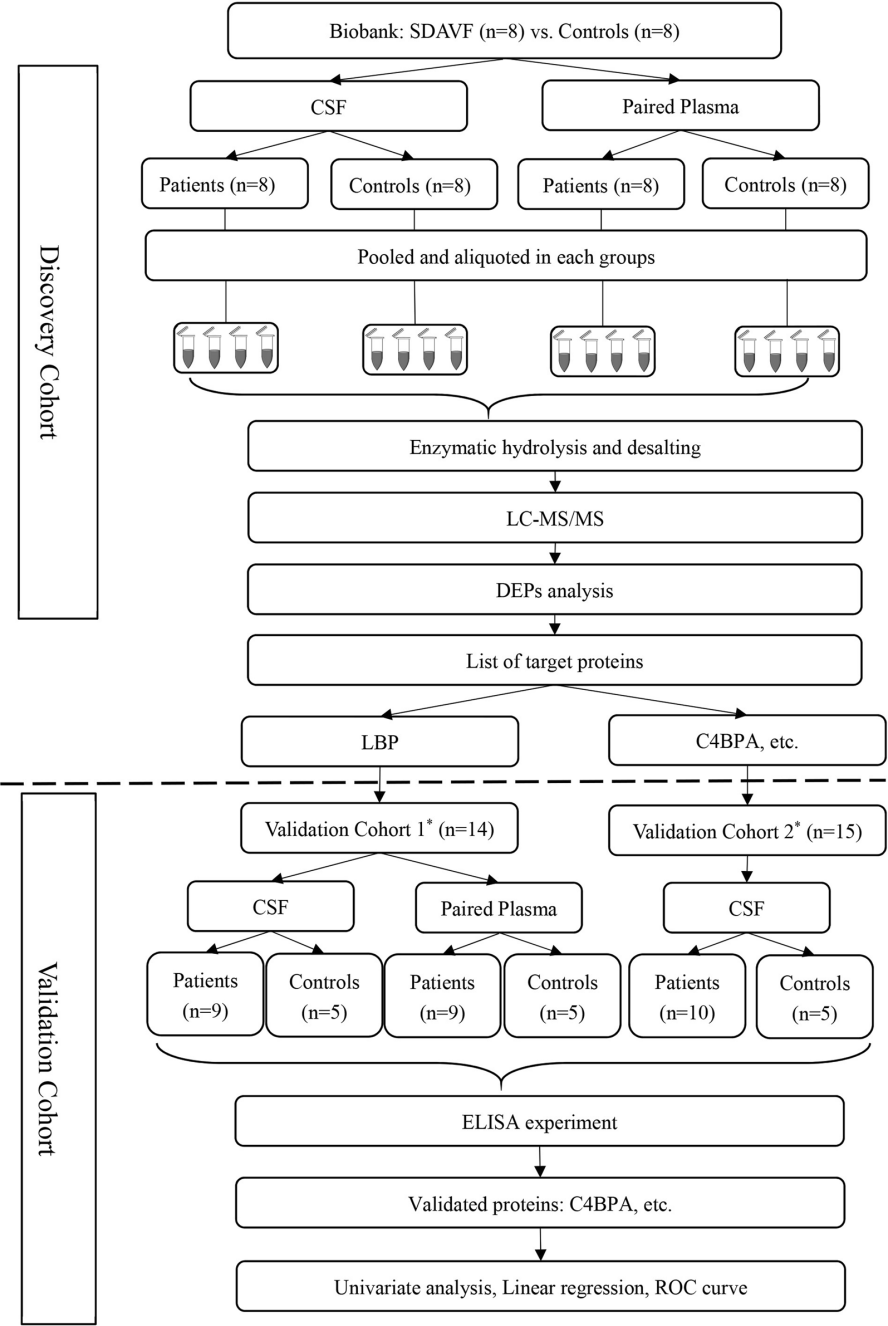
Study design flowchart. The discovery cohort was followed for at least 1 year, except one lost for the wrong contact detail. The validation cohort with at least a 6-month follow-up was divided into two cohorts due to the consumption of CSF samples in the exploration of experimental conditions. * The two validation cohorts had some duplicated patients (*n* = 2) and controls (*n* = 1) but were different from the discovery cohort

All participants were recruited at Capital Medical University Xuanwu Hospital from 2019 to 2020. The patient cohort was a part of the COPSDAVF study (*ClinicalTrials.gov*, NCT03192800), which is described in detail elsewhere [9]. In brief, patients who were diagnosed by DSA and received lumbar puncture before surgery were included in this study. The exclusion criteria were as follows: (1) ≤ 18 years of age; (2) history of lumbar surgery, stroke, demyelinating, and other neurological diseases; (3) upper cervical spinal cord or medulla oblongata oedema, incurring the risk of cerebral herniation; and (4) MRI or DSA revealed that the fistula drained into the horsetail, especially below the L2 vertebral level. Two researchers (Y. M. and C. Y.) independently screened clinical information to complete patient recruitment. We used modified Aminoff and Logue's Scale (mALS) [9] and modified Denis Pain and Numbness Scale (mDS) [10] to evaluate the patient's neurological function, and the combined scores (CS) were equal to mALS scores plus mDS scores (for the detailed items, see Additional file 2: Table S1). Scheduled follow-up was 3 months, 6 months, and 12 months. Patients were followed via outpatient visits or telephone interviews, and those in the validation cohort had at least 6 months of follow-up. The improvement of CS was calculated as follows: 6-month follow-up CS minus presurgical CS. One patient in the discovery cohort was lost to follow-up due to incorrect contact information. The control group included participants who underwent artificial joint replacement surgery by spinal anaesthesia with no history of neurological diseases. The sample size of the validation cohort was calculated as 10 in the patient group and 6 in the control group, with the hypothesis of AUC = 0.9. This study was approved by the Ethics Committee of Xuanwu Hospital and all subjects signed informed consent forms. The overall workflow is shown in Fig. 1.

### Sample preparation

Five to ten millilitres of cerebrospinal fluid (CSF)was collected by lumbar puncture. Two millilitres of CSF was sent to the hospital laboratory for routine examination including cell count and differential (XN-2000-Haematology-Analyser-Sysmex), total protein, and glucose (7600 Automatic Biochemical Analyser Hitachi). Meanwhile, paired 4 ml blood was collected using Vacutainer CPT tubes (BD Biosciences) containing EDTA. Samples were transferred to the biobank of our hospital at 4 ℃ within 2 h. Then, CSF and whole blood samples were centrifuged at 1500 r/min and 3000 r/min, respectively, for 10 min. The supernatant was collected in 0.5 ml aliquots in polypropylene tubes (Corning, 430,659) and stored at –80 °C.

### Protein digestion

In the discovery cohort, the 8 samples of each group were mixed and aliquoted into 4 centrifuge tubes (see Fig. 1). The mixed samples were lysed in buffer containing 4% sodium dodecyl sulfate and 0.1 M Tris–HCl (pH 7.6). The protein concentration was determined by the BCA protein assay kit (Thermo Scientific, Rockford, IL). The proteins (100 μg) were incubated with 100 mM DTT at 37 ℃ for 1 h. After incubation, the lysates were transferred to a centrifugal filter (Microcon YM-30, EMD Millipore Corporation, Billerica, MA), and replaced with 200 μl UA (8 M urea, 100 mM Tris. Cl pH 8.5) twice. After the buffer was replaced, proteins within UA were alkylated with 55 mM iodoacetamide (IAA, Sigma–Aldrich, Saint Louis, MO) in a dark at room temperature for 15 min. The UA buffer was subsequently replaced with 0.1 M triethylammonium bicarbonate (TEAB, Sigma–Aldrich, Saint Louis, MO) and digested with trypsin (Promega, Madison, WI) (1:50 (w: w)) at 37 ℃ overnight. The desalting of resultant tryptic peptides was conducted by StageTips and stored at − 20 °C.

### LC–MS/MS and data analysis

The peptides (2 μg) were resolubilized in 0.1% formic acid (FA) and analysed by a mass spectrometer (Orbitrap Fusion™ Lumos™ Tribrid™, Thermo Scientific, Rockford, IL Waltham, MA) coupled to an Easy-nLC 1200. The samples were run with mobile phases containing buffer A (0.1% FA) and buffer B (80% ACN, 0.1% FA). The peptides were separated by a capillary analytic C18 column (length: 25 cm, inner diameter: 150 μm, particle diameter: 1.9 μm) in a 180-min nonlinear gradient at a flow rate of 600 nl/min. For each three-second cycle, the full MS scan was acquired in the Orbitrap at a resolution of 120,000 with an automatic gain control (AGC) target of 5 × 10^5^, and higher-energy collisional dissociation (HCD, collision energy: 30%) was used to fragment these precursors. An MS/MS scan was performed in the IonTrap (AGC = 3 × 10^4^). MaxQuant (version 1.6) software was used for database search and label-free quantitative analysis. The Homo sapiens proteome sequence database was downloaded from the Uniprot website [11]. The parameters of the database search were set as follows: type: standard; multiplicity: 1; the protease used for protein digestion: trypsin; label-free quantification: LFQ; the minimum score for unmodified peptides: 15. All other parameters were default values. The mass spectrometry proteomics data have been deposited to the ProteomeXchange Consortium via the PRIDE [12] partner repository with the dataset identifier PXD030479.

### Bioinformatics analysis

The data analysis of this data was mainly run in R (version 3.6.3). We defined significantly differentially expressed proteins (DEPs) as those with a fold change > 1.5 (up- and down-regulated) and *P* < 0.05. The DEPs were visualized by volcano plot, cluster heatmap, and Venn plot via the ggplot2 package. The annotation and enrichment of GO terms and KEGG pathways was achieved through the clusterProfiler package and org.Hs.e.g.db package [13]. The protein–protein interaction (PPI) annotation of DEPs was obtained from the STRING database (Version 11.5, string-db.org). The PPI subcluster and top10 hub genes were extracted via MCODE and the CytoHubba plugin of Cytoscape software (version 3.8.2).

### ELISA assessment

Seventeen cases and nine controls were included in the validation cohort. The experimental performers (Z.S. and A.T.) were blinded to group information. Sources of the ELISA kit and dilution fold of CSF samples were listed as follows: LBP (2× dilution; Human, E-EL-H6108, elabscience, China), APP (2× dilution; Human, E-EL-H1216c, elabscience, China), C4BPA (2× dilution; Human, CSB-E11170h, cusabio, China), APOB (200× dilution; Human, E-EL-H0464c, elabscience, China), C1QA (10× dilution; Human, CSB-EL003637HU, cusabio, China), C4 (50× dilution; Human, CSB-E08705h, cusabio, China), and MASP2 (Neat; Human, CSB-E17966h, cusabio, China). All samples were set in triplicate wells. According to the manufacturer’s manual, standards or samples (100 µl each) were added to 96-well plates and incubated for 90 min at 37 °C. After discarding the liquid from each well, a biotinylated antibody working solution (100 µl) was added to each well and incubated at 37 °C for 1 h. The plates were washed 3 times. Subsequently, 100 μl of the horseradish peroxidase (HRP)-streptavidin conjugate was added to each well and incubated for 30 min at 37 °C. The plates were then washed 5 times, and 90 μl of substrate solution was added to each well and incubated at 37 °C in the dark for 15 min. The reaction was stopped by adding 50 µl of stop solution and the absorbance value was immediately measured at 450 nm (Multiskan FC, Thermo Scientific).

### Statistical analysis

All statistical analyses and visualization were performed in R (version 3.6.3) or GraphPad Prism (version 8.0.1). The sample size was estimated by PASS (version 15.0.5). The quantitative results of ELISA were normalized to the total protein in each patient. An independent samples t test was used to compare the means of continuous normally distributed data. The Chi-squared test was adopted for the comparison of the categorical variables, such as sex. When the data showed a non-normal distribution, Wilcoxon rank-sum tests were applied. The association of target proteins with the clinical factors was visualized by scatter plot and fitted by simple linear regression. Receiver operating characteristic (ROC) curves were generated to investigate the diagnostic performance of biomarkers. All statistical tests were two-tailed. *p* or adj. *p* < 0.05 indicated statistical significance.

## Results

### Diagnosis and prognosis of SDAVF are intractable problems

**Fig. 2:**
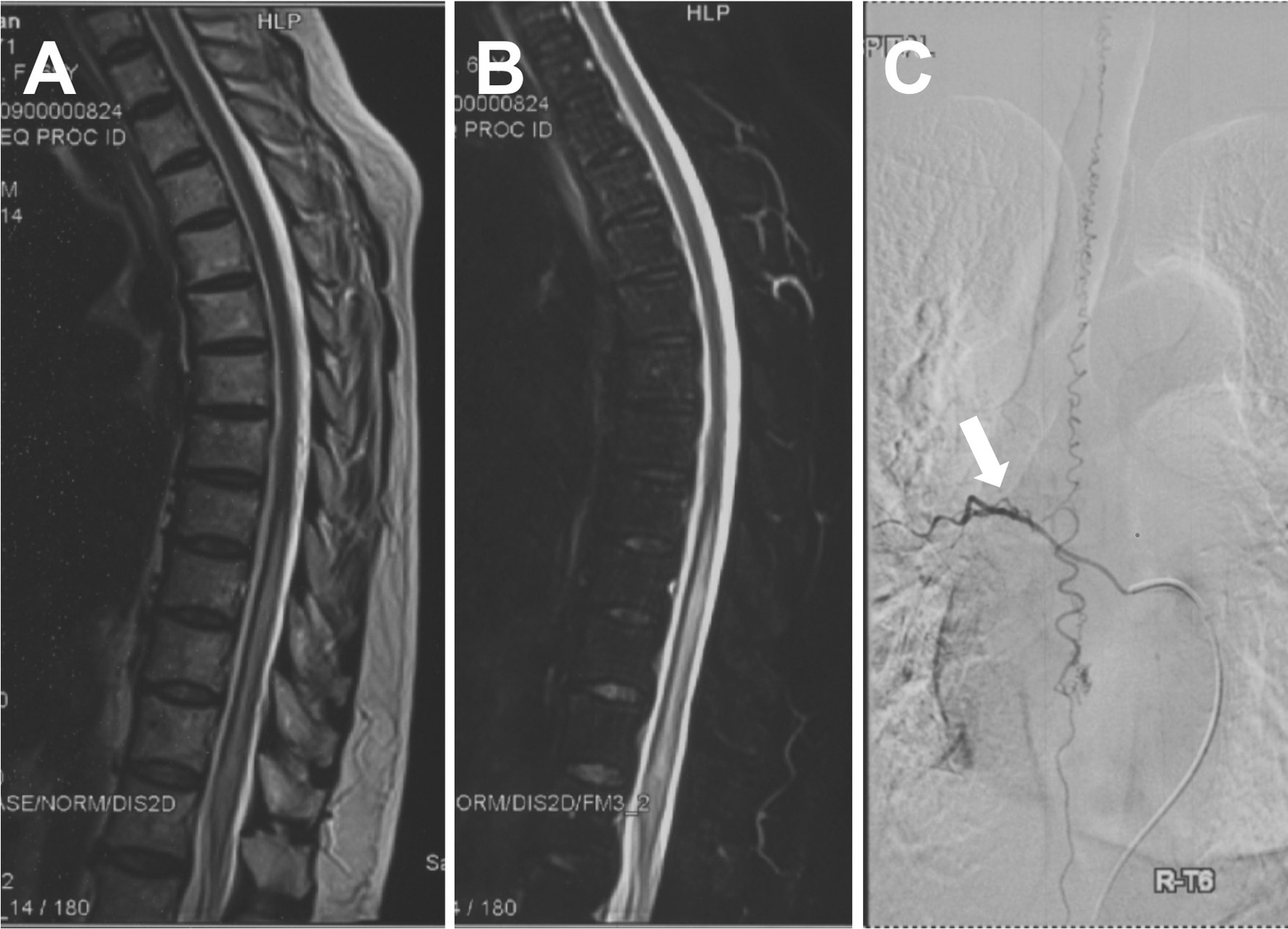
Imaging findings of a patient with misdiagnosis. A 69-year-old woman complained of lower-extremity progressive weakness and numbness over one year. Magnetic resonance imaging showed a multisegmental intramedullary T2 hyperintensity (**A**) with diffuse contrast enhancement (**B**). There was no evidence of obvious fluid voids. **C** DSA revealed a fistula (white arrow) at the right T6 level, fed by the radicular artery rising from the segmental artery

A total of 25 patients and 17 controls were included in this study. Clinical features of the patients were reported at the 1-year (discovery cohort) and 6-month (validation cohort) follow-ups, as shown in Table 1. The mean age of the patients was 56.8 ± 11.7 years, with a male-to-female ratio of 4:1. The median duration was 10 months (interquartile range, 6 to 14 months). The total protein of CSF was elevated slightly in approximately 60% of patients. Five patients (20%) suffered sudden paraplegia during the steroid therapy procedure in the local hospital, in which two patients revealed no obvious fluid-void sign on MRI (see Fig. 2). All patients underwent microsurgery operations through a posterior midline approach to occlude the fistula and no recurrence was found at the end of follow-up. Although all of the patients achieved anatomical healing on restaging MRI or DSA, four cases (16%) had symptoms that worsened. Seventy to eighty percent of patients had symptomatic improvement at the end of follow-up, but only one had a complete recovery to normal. The individual recovery rate was approximately 30% at the end of follow-up, which indicated residual disability in most patients. In addition to the sex difference in validation cohort 2 (*p* = 0.0256), no obvious differences in baseline were found between patients and controls, as shown in Additional file 3: Table S2.

**Table 1:** Clinical characteristics of the SDAVF patients

	Discovery cohort (*n* = 8)	Validation cohort (*n* = 17)
Age (mean ± SD, year)	51.6 ± 14.9	59.3 ± 9.4
Sex (male, %)	75.0%	78.9%
Disease duration (median + IQR, Mon.)^§^	14.6 ± 9.5	9.9 ± 7.0
History of steroid therapy^†^	25.0%	17.6%
Total protein of CSF (mean ± SD, mg/dl)	–	56.1 ± 24.5
*MR imaging (positive, %)*		
Long T2 signal	75.0%	64.7%
Fluid-void	87.5%	94.1%
*Fistula location by DSA*		
Cervical	–	5.9%
Thoracic	87.5%	70.5%
Lumbar	12.5%	23.6%
*Combined score (CS, Mean ± SD)*		
Preoperation	8.8 ± 1.4	9.6 ± 3.5
Hospital discharge	8.5 ± 2.0, *p*^‖^ = 0.5983	8.6 ± 3.0, *p*^‖^ = 0.0370
The last follow-up^Ø^	7.1 ± 3.8, *p*^‖^ = 0.1428	6.4 ± 3.4, *p*^‖^ = 0.0068
Worsen rate (%)^£^	12.5%	17.6%
Improvement rate (%)^¥^	71.4%	76.5%
Recovery rate (median + IQR, %)^$^	36.4% (3.6%, 50%)	15.6% (− 3.1%, 46.0%)

§: duration of the disease was the time from onset to last hospitalization. †: A total of five patients suffered acute exacerbation after the corticosteroid therapy, which varied in class, dose, and duration, but drug withdrawal occurred over 1 month before the last hospitalization. Ø: The last follow-up was 1 year in the discovery cohort and 6 months in the validation cohort. ‖: A two-tailed paired t test was performed to compare the scores pre- and postoperation. £: "Worsen" was defined as the follow-up CS increasing over 1 point compared to the preoperative score. ¥: "improvement" was defined as the follow-up CS decreasing over 1 point compared to the preoperative level. $: the individual recovery rate (%) = (preoperative CS − postoperative CS)/preoperative CS × 100%. IQR: interquartile range. "-" refers to no data available

### Complement activation within the CSF was the pathophysiological feature of VHM

**Fig. 3:**
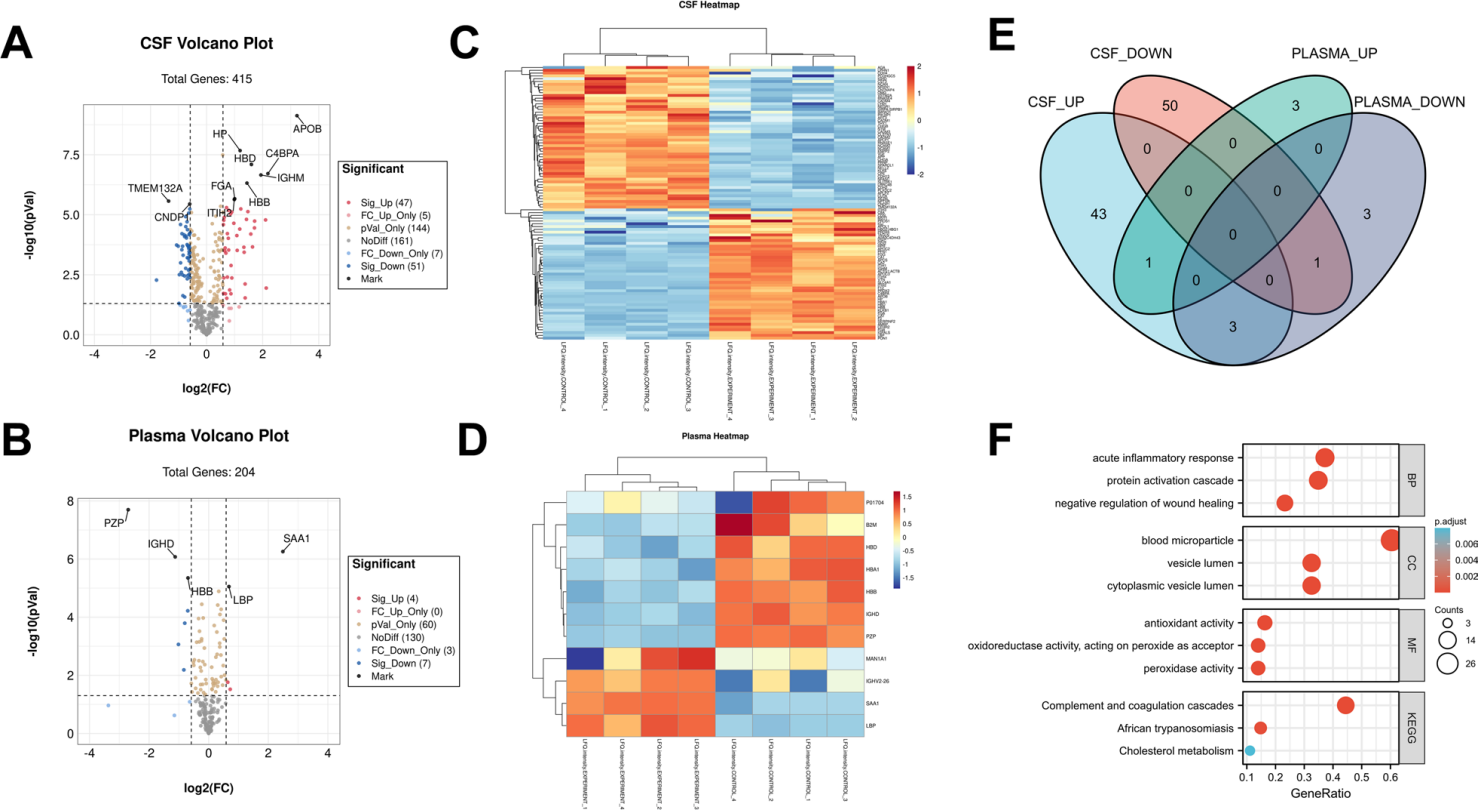
Preliminary analysis of DEPs. Volcano plot of CSF (**A**) and paired plasma samples (**B**) comparing SDAVF patients with the controls. The red dots refer to significantly overexpressed proteins, and significantly downregulated proteins are coloured blue. Cluster heatmap of CSF (**C**) and paired plasma samples (**D**) comparing SDAVF patients with controls. The ‘red’ blocks refer to overexpression and downregulated proteins are coloured ‘blue’. The colour intensity indicates the degree of fold change. **E** The Venn diagram summarizes the relationship between the two datasets. **F** GO term/KEGG pathway enrichment analysis of overexpressed proteins in CSF samples. The size of the nodes denotes the number of proteins, and the colour represents the adjusted *p* value

In the discovery cohort, a total of 98 differentially expressed proteins (DEPs) were identified from CSF samples, and 11 DEPs appeared in plasma samples (Fig. 3A–D, Additional file 4: Table S3). APOB and C4BPA were the most differentially expressed proteins in CSF samples with fold changes of 9.34 and 4.55, respectively. We next found some overlap between the CSF and plasma datasets (Fig. 3E). LBP was upregulated in both datasets, whereas HBD, HBA1, and HBB were upregulated in patients’ CSF and downregulated in their plasma. To characterize the biological pathways associated with the DEPs, we performed GO and KEGG enrichment analyses on CSF samples. The GO analysis results showed that the upregulated proteins were mainly enriched in the acute inflammatory response, protein activation cascade, blood microparticles, and antioxidant activity. KEGG enrichment showed that the complement and coagulation cascades pathway was the most overexpressed pathway, and approximately 40% (12/27, including C4BPA and C8A) of molecules in this cluster were up-regulated (Fig. 3F, details in Additional file 5: Table S4).

### Enrichment of the PPI network revealed important role of the complement pathway

**Fig. 4:**
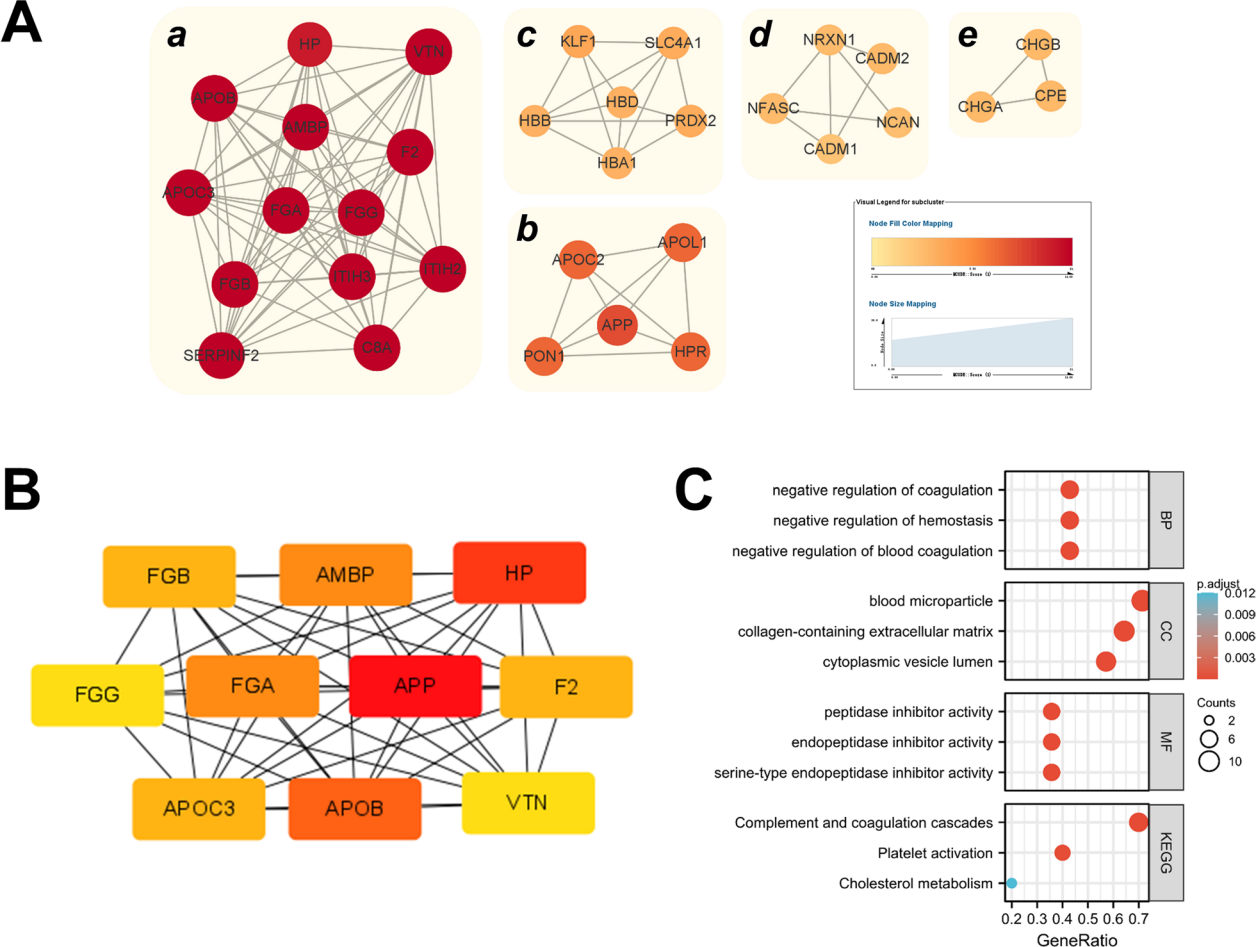
PPI network and hub gene analysis. Five interactional subclusters (**A**) and the top 10 hub genes (**B**) were obtained from 98 DEPs in CSF samples. The node score cut-off was set as 0.2. Continuous colour variation from deep to shallow reveals the score (**A**) or rank (**B**) from high to low. **C** Enrichment and visualization of GO/KEGG annotation based on the subclusters and hub gene list. The size of the nodes denotes the number of proteins, and the colour represents the adjusted *p* value

The 98 DEPs in CSF samples were enriched into 5 highly interconnected subclusters by Cytoscape software (Fig. 4A). The highest rating subcluster included 76 edges and 13 nodes, involving APOB, C8A, etc. Subsequently, the hub genes of the PPI network were screened by the degree method. APP (fold change = 0.59), HP, and APOB were the top three hub genes in the list (Fig. 4B). Finally, we picked up all 23 key proteins from the highest rating subcluster and the hub gene list to perform GO/KEGG analysis. The results demonstrated that the complement and coagulation cascade pathways were significantly enriched, which was consistent with previous analysis (Fig. 4C).

### C4BPA and C1QA might be potential diagnostic biomarkers

**Fig. 5:**
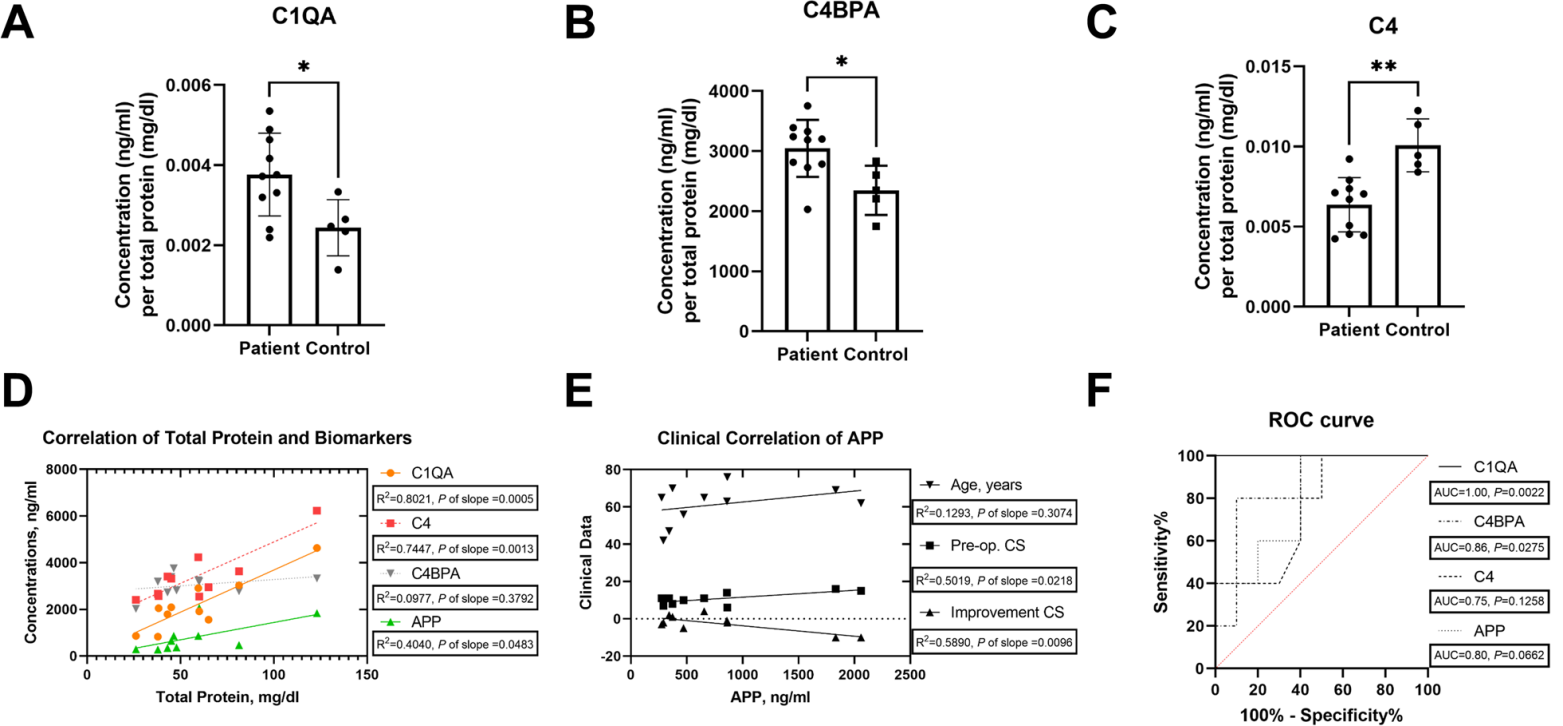
Quantitative analysis and clinical relevance of potential biomarkers. **A**–**C** The specific protein levels were measured in CSF samples via ELISA kits. The concentration (*Y*-axis) was normalized to the total protein in CSF. Generally, data that passed the normality test were described by the mean with SD and compared by unpaired *t* test. *p < 0.05, “ns” refers to *p* > 0.05. **D**, **E** Simple linear regression was used to assess the correlation between clinical factors and proteins levels. Solid lines are the fit of linear regression, and dotted lines represent the 95% confidence interval. **F** ROC analysis of multiple variables. The diagonal dashed line reflects a random prediction (AUC = 0.5)

Considering the above results, we prudently chose LBP, APOB, APP, C4BPA, C4, C1QA, and MASP2 to validate feasibility for diagnosis. The ELISA results demonstrated high levels of C1QA and C4BPA in patient’s CSF (Fig. 5A, B, average 2167.2 ng/ml and 3046.9 ng/ml, respectively). Although the proportion of C4 was decreasing in the patient’s CSF (Fig. 5C, *p* = 0.0015), the absolute quantitative level (Mean, 3396.1 ng/ml; SD, 1091.8 ng/ml) was slightly higher than that in the control group (*p* = 0.1587). Regarding other biomarkers, including APP, LBP, APOB and MASP2, no significant differences were found between the two groups (Additional file 1: Figure S1). Additionally, the correlation between biomarkers and clinical variables was assessed. The levels of C1QA, C4, and APP were positively correlated with the total CSF protein level (Fig. 5D). Moreover, APP showed a positive correlation with preoperative CS (*R*^2^ = 0.5019, *p* = 0.0218) and a negative correlation with postsurgical neurological function improvement (*R*^2^ = 0.5890, *p* = 0.0096, Fig. 5E). Remarkably, there were two extremely high values in the APP examination patients group (Additional file 1: Figure S1). Common clinical features that differed from the others were not present in the two patients, with the exception of highest preoperative CS scores. Hence, a sensitivity analysis was conducted for Fig. 5D, E by excluding the two patients. The results showed that linear correlation was almost retained in C1QA (*R*^2 ^= 0.5459, *p* = 0.0363) and C4 (*R*^2^ = 0.4254, *p* = 0.0796) with total protein, while APP was nonsignificantly correlated with clinical characteristics. Finally, we plotted a receiver operating characteristic (ROC) curve to assess the diagnostic efficacy of C1QA, C4BPA, C4, and APP (Fig. 5F). The area under the curve of C1QA and C4BPA was 1.00 and 0.86, respectively (*p* < 0.05). When the threshold of C1QA was 787.2 ng/ml, both the sensitivity and specificity were 100% (95% CI, 56.55%–100%, 72.25%–100%, respectively).

## Discussion

The initial stage of SDAVF is insidious and easily misdiagnosed because symptoms are similar to those of myelitis. Many case series have suggested that steroid therapy can exacerbate symptoms [14]. Kaut et al. reported a case with a 6-year duration. The patients stayed in bed due to complete paralysis after treatment with steroid therapies. Although the fistula was cut off completely via surgical operation, lower limb strength did not fully recover. It has been widely accepted that corticosteroids increase the retention of salt and water, leading to venous volume overload and worsening of spinal oedema [15]. In this study, 20% of patients had a history of steroid therapies, which emphasized the importance of accurate diagnosis.

The current screening strategy for SDAVF mainly relies on magnetic resonance imaging with or without enhancement. Research has verified the fluid-void sign as a diagnostic marker with higher specificity (almost 100%), but its sensitivity was only approximately 80% [7, 16]. Lindenholz et al. proposed that magnetic resonance angiography (MRA) might be an effective diagnostic approach with 78% specificity and 91% sensitivity [17]. In 2018, Zelawski et al. reviewed MR enhancement data of 44 SDAVF patients at Mayo Clinic over the past 20 years, and founding that 44% of patients had a “missing-piece sign” [18]. Although DSA was generally accepted as a gold standard for SDAVF diagnosis, they also found some patients confirmed until the second or third angiogram. Therefore, a combination screening strategy including laboratory examinations was necessary. Some scholars have summarized a nonspecific change in CSF of SDAVF, such as slightly increased albumin and cell counts [19]. This study is the first proteomic analysis of SDAVF biomarkers. We found that C4BPA and C1QA were overexpressed in the CSF of patients, which was validated in an independent cohort. Furthermore, their concentration was positively correlated with the CSF total protein. In the ROC analysis, it is conceivable that C1QA and C4BPA have diagnostic potential with AUC values of 1.00 and 0.86, respectively.

To date, the aetiology of SDAVF is still unclear, and it is generally accepted to be an acquired disease. Some scholars claim it could be induced by surgical operation and trauma [20]. Jellema et al. found a small physiological shunt between the radicular artery and venous plexus through a postmortem study, indicating that the environmental factors might lead to disease onset [21]. Due to the low incidence rate and difficulty of clinical biopsy, there is a relative lack of research on its pathogenesis. Previous studies have shown that VHM and spinal cord necrosis are the pathophysiological processes of SDAVF [22, 23]. The VHM contributed to arterialization of venous drainage, hyalinization of the microvasculature, vascular calcification, and thrombosis, which in turn caused neuron necrosis, glial hyperplasia, and macrophage infiltration. These pathophysiological changes were also verified in our previous study in a rabbit model, but the specific molecular mechanism remains to be explored further [24]. Recently, Liu et al. revealed that the draining vein of SDAVF had artery-like structures and blood–brain barrier (BBB) disruption, accompanied by endothelial injury, CD45 + cell infiltration, and COX-1 overexpression. Here, we found abnormal activation of the complement and coagulation cascade pathways in the patient’s CSF. PPI network analysis and hub gene screening confirmed the reliability of this pathway. These results implied that complement activation plays an important role in the processes of venous hypertensive myelopathy.

As an important effector of the immune response, the complement system consisting of over 40 proteins could be activated by the classical, lectin, and alternative pathways. Whatever the activation mechanism, three major effector functions result: inflammatory cell recruitment, opsonization, and cell lysis. Increasing evidence has revealed the wide endogenous expression of complements in the central nervous system [25]. In this study, activation of the complement system was not found in peripheral blood, and the normalized concentrations of C1QA and C4BPA were elevated in patient CSF, which supported the intrathecal synthesis of complement proteins. Although the lectin pathway was enriched in KEGG analysis, it included a fair number of coagulation factors and fibrinogens (Additional file 5: Table S4). We speculated that this might be affected by damage to the BBB. Further ELISA experiments validated this point. As the upstream molecule generating C3 convertase (C4b2a), C1Q of the classical pathway was overexpressed, but MASP2 of the lectin pathway was not. Therefore, we thought that the classical complement activation (not the lectin pathway) and BBB disruption might coexist in the central nervous system of SDAVF patients. On the other hand, the C1 complex (involving C1q, C1r2, and C1s2) and MASP1/2 cleave C4 and C2 into fragments to form the C3 conversion enzyme (C4b2b) [25]. C4BP, a regulatory protein of the C3 conversion enzyme, was important both in the formation of the membrane attack complex (MAC) and enhancement of macrophage function [26]. In this study, we found that hyperactivation of the complement system might drive the compensatory elevation of C4BPA and relative depletion of C4. Additionally, there was no difference in C5a expression compared to controls (Additional file 4: Table S3), which indicated that recruitment of inflammatory cells was not the main function of complement activation in the VHM [26]. This finding is also consistent with the result that almost normal cell counts were observed in the laboratory examination [19]. Hence, we assumed that the complement cascade driven by the classical pathway tended to result in phagocytosis of microglia, rather than cell lysis or inflammatory recruitment.

This study also had some limitations. Due to the limited samples and the exploratory nature of the study, individual or batch heterogeneity might have affected the outcome of APOB, LBP and APP. Further studies with more patients and longer follow-up times are needed to refine our findings. Several studies have shown that both Alzheimer's disease and spinal cord injury are associated with the complement cascade [25, 27]. Subsequent research should include disease control groups, such as transverse myelitis and multiple sclerosis, to improve differential diagnosis. Finally, SDAVF was more common in the males, while sex differences were obvious in validation cohort 2. Studies have shown that males with reproductive years (ages 20–50) had high levels of complement proteins, but sex differences in inflammatory molecules were not significant in elderly patients [28, 29]. The results of this study might be interpreted with caution, although the mean age of all subjects in validation cohort 2 was over 60 years.

## Conclusions

Overall, for the first time, our study identified C4BPA and C1QA as potential biomarkers for the diagnosis of SDAVF. The classical pathway of complement activation might be one of the molecular mechanisms for venous hypertensive myelopathy.

## Supplementary Information

**Additional file 1: Figure S1.** Negative results of quantitative analysis.

**Additional file 2: Table S1.** Clinical rating scales of the study.

**Additional file 3: Table S2.** The comparison of baseline characteristics.

**Additional file 4: Table S3.** The result of protein quantification and differential analysis.

**Additional file 5: Table S4.** The result of GO/KEGG enrichment in CSF sample.

## Data Availability

The datasets generated and analysed during the current study are available in the ProteomeXchange Consortium (http://proteomecentral.proteomexchange.org). Information on the target proteins and proteome sequences were obtained from a third-party public database with correct citations.

## Abbreviations

SDAVF: Spinal dural arteriovenous fistula
VHM: Venous hypertensive myelopathy
CSF: Cerebrospinal fluid
mALS: Modified Aminoff and Logue's Scale
mDS: Modified Denis Pain and Numbness Scale
CS: Combined scores
DEPs: Differential expressed proteins
GO: Gene ontology
KEGG: Kyoto Encyclopedia of Genes and Genomes
PPI: Protein–protein interactions
ELISA: Enzyme-linked immunosorbent assay
MR: Magnetic resonance
DSA: Digital subtraction angiography
LC–MS/MS: Liquid chromatography–tandem mass spectrometry
ROC: Receiver operator characteristic
AUC: Area under the curve
C4BPA: Complement component 4 binding protein alpha
APP: Amyloid beta precursor protein
APOB: Apolipoprotein B
LBP: Lipopolysaccharide binding protein
BBB: Blood–brain barrier
