# Supplementary material for: Potential biomarkers of spinal dural arteriovenous fistula: C4BPA and C1QA
Source: J Neuroinflammation. 2022 Jun 22;19:165. doi: 10.1186/s12974-022-02522-x (PMC9215050; doi:10.1186/s12974-022-02522-x)
Supplement: Supplementary file 2 — Additional file 2: Table S1. Clinical rating scales of the study. [file 12974_2022_2522_MOESM2_ESM.docx]

| Gait |  |
| --- | --- |
| 0 | Normal leg power, stance and gait |
| 1 | Leg weakness with no restriction of walking |
| 2 | Restricted exercise tolerance |
| 3 | Requires one stick or some support for walking |
| 4 | Requires crutches or two sticks for walking |
| 5 | Requires a wheelchair |
| Urination |  |
| 0 | Normal |
| 1 | Urgency, frequency and/or hesitancy |
| 2 | Occasional incontinence or retention |
| 3 | Persistent incontinence or retention |
| Defecation |  |
| 0 | Normal |
| 1 | Mild constipation, responding well to apperients |
| 2 | Occasional incontinence or persistent constipation |
| 3 | Persistent incontinence |

Table 1 Modified Aminoff and Logue Scale

Table 2 Modified Denis Scale

| Numbness |  |
| --- | --- |
| 1 | None |
| 2 | Mild and occasional numbness |
| 3 | Moderate numbness, not impacting on daily life and work |
| 4 | Persistent severe numbness, impacting the quality of life |
| Pain |  |
| 1 | None |
| 2 | Mild and occasional pain, without medication |
| 3 | Moderate pain with medication occasionally, not impacting on daily life and work |
| 4 | Moderate pain with medication frequently, impacting on daily life and work |
| 5 | Persistent severe pain requiring daily meditation |
